# hCG: Biological Functions and Clinical Applications

**DOI:** 10.3390/ijms18102037

**Published:** 2017-09-22

**Authors:** Chinedu Nwabuobi, Sefa Arlier, Frederick Schatz, Ozlem Guzeloglu-Kayisli, Charles Joseph Lockwood, Umit Ali Kayisli

**Affiliations:** 1 Department of Obstetrics & Gynecology, Morsani College of Medicine, University of South Florida, Tampa, FL 33612, USA; sefaarlier@gmail.com (S.A.); fschatz@health.usf.edu (F.S.); ozlem2@health.usf.edu (O.G.-K.); cjlockwood@health.usf.edu (C.J.L.); 2 Department of Obstetrics & Gynecology, Adana Numune Training and Research Hospital, Adana 01370, Turkey

**Keywords:** human chorionic gonadotropin (hCG), α and β hCG subunits, luteinizing hormone/chorionic gonadotropin receptor (LHCGR), trophoblasts, pregnancy, clinical applications

## Abstract

Human chorionic gonadotropin (hCG) is produced primarily by differentiated syncytiotrophoblasts, and represents a key embryonic signal that is essential for the maintenance of pregnancy. hCG can activate various signaling cascades including mothers against decapentaplegic homolog 2 (Smad2), protein kinase C (PKC), and/or protein kinase A (PKA) in several cells types by binding to luteinizing hormone/chorionic gonadotropin receptor (LHCGR) or potentially by direct/indirect interaction with transforming growth factor beta receptor (TGFβR). The molecule displays specialized roles in promoting angiogenesis in the uterine endothelium, maintaining myometrial quiescence, as well as fostering immunomodulation at the maternal-fetal interface. It is a member of the glycoprotein hormone family that includes luteinizing hormone (LH), thyroid-stimulating hormone (TSH), and follicle-stimulating hormone (FSH). The α-subunit of hCG displays homologies with TSH, LH, and FSH, whereas the β subunit is 80–85% homologous to LH. The hCG molecule is produced by a variety of organs, exists in various forms, exerts vital biological functions, and has various clinical roles ranging from diagnosis and monitoring of pregnancy and pregnancy-related disorders to cancer surveillance. This review presents a detailed examination of hCG and its various clinical applications.

## 1. Introduction

In early pregnancy, human chorionic gonadotropin (hCG) is produced primarily by differentiated syncytiotrophoblasts, and represents a key embryonic signal [1,2] essential for thre maintenance of pregnancy. During the initial six weeks of pregnancy, hCG promotes secretion of progesterone, estradiol, and estrone via transformation of the post-ovulatory ovary into the gravid corpus luteum [3]. Furthermore, hCG binds to its receptor to perform specialized roles in promoting angiogenesis in the uterine endothelium [4], maintaining myometrial quiescence [5], as well as fostering immunomodulation via alteration of activity of dendritic cells, the reduction of T-cell activation and cytokine production, promotion of T regulatory (Treg) cell recruitment, and an increase in proliferation of uterine natural killer (NK) cells at the maternal-fetal interface [6,7]. Metabolism of hCG by the placenta, liver, blood, and kidney determines its steady-state levels [8,9]. Measurements of serum or urine hCG levels provide important information in a variety of clinical situations, such as diagnosis and monitoring of pregnancy and pregnancy-related disorders, prenatal screening, and gynecological cancers [10]. This review focuses on classification, functions, and clinical diagnostic and therapeutic applications of hCG.

## 2. Structure, Isoforms, and Genes of Human Chorionic Gonadotropin (hCG)

As a 237 amino acid heterodimer, hCG is comprised of α-(93-amino acid, 14.5 kD) and β-(145-amino acid, 22.2 kD) subunits that are non-covalently linked by charge interactions and contain a total of eight carbohydrate side chains [11]. It is a member of the glycoprotein hormone family that includes luteinizing hormone (LH), thyroid-stimulating hormone (TSH), and follicle-stimulating hormone (FSH). The α-subunit of hCG is homologous to TSH, LH, and FSH, whereas the β-subunit is 80–85% homologous to LH [10,12]. Specifically, the LH β-subunit contains 121 amino acids, whereas hCG β-subunit consists of 145 amino acids. The 24 amino acid difference between the hCG and LH β-subunits, which comprises amino acids 121–145, is unique to hCG and referred to as the C-terminal peptide (CTP) [10,12,13]. Consequently, several hCG antibodies also recognize LH, and vice versa [12]. As a result of its structural homogeneity to LH, hCG binds to luteinizing hormone/chorionic gonadotropin receptor (LHCGR) during the first 3–4 weeks of pregnancy, stimulating corpus luteal cells until the steroidogenic activity of the placenta produces sufficient progesterone to maintain pregnancy [14,15,16].

The eight carbohydrate side-chains in the hCG structure account for 30% of its molecular weight. Specifically, hCGα contains two N-linked carbohydrate chains on Asn52 and Asn78, whereas hCGβ contains two N-linked glycans at Asn13 and Asn30, and four O-linked glycans linked to Ser121, Ser127, Ser132, and Ser138 [12]. The secretion, half-life, and functions of hCG all depend on its glycosylated state. Specifically, the sialic (*N*- or *O*-substituted derivatives of neuraminic acid) content of hCG plays vital roles in its receptor binding affinity, function, and clearance from the circulation [17]. Post-translational modifications of hCG result in three dimeric isoforms: “regular” hCG, sulfated hCG (hCG-S), and hyperglycosylated hCG (hCG-H). The pituitary is the sole source of hCG-S secretion. During the menstrual cycle, hCG-S mediates several endocrine functions by inducing theca cell androstenedione production, corpus luteal progesterone production and by contributing to the process of ovulation. During pregnancy, syncytiotrophoblast derived hCG also induces corpus luteal cells to produce progesterone, whereas cytotrophoblast-derived hCG-H appears to act as an autocrine and paracrine factor by activating the transforming growth factor-β receptor (TGFβR) mediated signaling [18,19,20,21]. The hCG-H produced early in pregnancy and by various cancers contains more structurally-complex sialylated glycans than hCG-H produced in mid and late pregnancy [10,22,23].

The hCG α-subunit is encoded by a single gene (*CGA*) localized to chromosome 6q21.1-23 [24] while the β-subunit is encoded by six non-allelic genes (*CGB1*, *2*, *3*, *5*, *7* and *8*) localized to chromosome 19q13.3 [25,26]. This gene cluster is widely accepted to have evolved as a result of duplication from the *LHB* gene [1] resulting in their highly conserved structure at the nucleotide level (85–99% DNA sequence identity) [27]. Expression of hCG genes is regulated by several hormones (corticosteroids, progesterone, GnRH), growth factors (placental growth hormone, leukemia inhibitory factor, vascular endothelial growth factor (VEGF)), cytokines (Interleukin (IL)-6, epidermal growth factors (EGF), tumor necrosis factor (TNF)-α), ligands of the nuclear receptor PPARγ and the homeobox gene (DLX3) [28,29,30,31].

## 3. Role of hCG in Embryo Implantation and Trophoblast Invasion

The process of embryo implantation occurs approximately eight to 10 days after ovulation [32] and involves a series of complex steps including: (1) apposition of the blastocyst at the endometrial surface; (2) initial adhesion of the blastocyst to the endometrium; (3) convergence of trophoblast microvilli with pinopodes, also known as micro-protrusions from the apical end of the uterine epithelium; (4) trophoblast migration through the endometrial surface epithelium; (5) cytotrophoblast invasion of the decidua, i.e., the pregnant endometrial stroma, followed by localized disruption of endometrial capillary beds; and (6) remodeling of the vascular bed and formation of trophoblastic lacunae [33,34,35]. As a result of these sequential steps, the blastocyst is completely embedded in the decidua by day 10 [36,37]. This process is accompanied by fusion of cytotrophoblasts resulting in syncytiotrophoblast formation, also known as syncytialization, which continues throughout pregnancy.

Binding of hCG to LHCGR activates adenylate cyclase, phospholipase C and ion channels which, in turn, control levels of intracellular cAMP, inositol phosphates, Ca^2+^, and regulate activity of other second messengers [38,39,40]. Subsequently, cAMP acts via protein kinase A (PKA) to promote cytotrophoblast fusion and microvilli formation with both actions essential for protein secretion and nutrient/gas exchange by the resulting syncytiotrophoblasts [41]. Moreover, studies demonstrated hCG activation of protein kinase B (AKT) and ERK1/2 MAPK signaling in various cell types expressing LHCGR including COS-7 cells (kidney cell line from African green monkey), HGL5 cells (Human Granulosa Cell line) and primary human granulosa cells [39]. A recent study also demonstrated that in the HEK293 (Human embryonic kidney) cell line transiently co-expressing LHCGR and β-arrestin 2, recombinant hCG induces β-arrestin 2 recruitment to LHCGR in a concentration-dependent manner. This finding suggests involvement of β-arrestins in modulation of G-protein mediated signaling by hCG-LHCGR interaction since β-arrestins play crucial roles not only in desensitization/internalization of G-protein-coupled receptors (GPCRs) but also in their signaling as well as G-protein independent activation of ERK1/2 cascade [42]. Future studies are necessary to confirm hCG-mediated modulation of these various signaling cascades in primary trophoblast cultures.

Studies of in vitro fertilization (IVF) cycles detected secreted hCGβ in the culture medium as early as the time of transfer of eight-cell stage embryos, which occurs two days after fertilization, whereas hCG was not detected until eight days after egg retrieval [43]. Therefore, the increase in hCG levels between days 5 and 9 after ovum collection primarily reflects production of free hCGβ, whereas by day 22 circulating hCG consists predominately of α and β heterodimers [43]. Alternatively, in addition to hCGβ, other glycosylated hCG isoforms or truncated hCG not detected by current assays may be produced as early as day 5 and thereafter. This may result in biologically-active hCGβ via heterodimerization with other isoforms and binding to the receptor.

The impact of hCG on embryo implantation was recently investigated using in vitro models and ex vivo studies in humans. Incubation of cultured endometrial epithelial cells with recombinant hCG up-regulates production of implantation promoting factors such as leukemia inhibitory factor (LIF), prokineticin 1, VEGF, IL-11, CX3CL1, CCL14, and CCL4 [44,45,46]. Use of LIF knockout mice demonstrated that LIF is required for blastocyst implantation. This conclusion is derived from experiments demonstrating that females lacking a functional LIF gene produce fertilizable oocytes that are able to develop to the pre-implantation stage, but fail to implant [47]. The blastocyst also actively participates in implantation by releasing pro-implantation factors such as hCG [48]. Several other factors derived from maternal or embryonic cells, such as integrins, mucins, L-selectin, cytokines, as well as proteinases have been documented to play a crucial role in embryo implantation [35,49].

During the initial eleven weeks of pregnancy, invasive extravillous trophoblasts (iEVTs) actively secrete hCG dominated by hCG-H [19,30,50,51]. Accordingly, in maternal blood, hCG-H is elevated early in the first trimester corresponding to trophoblast invasion of decidua and then decreases. In addition to its endocrine function, iEVT-derived hCG-H acts as an autocrine factor that promotes iEVT invasion [1,48]. The specific receptor(s) activated by hCG-H on trophoblasts cells and, potentially, on various decidual cells has/have not been fully identified. However, using various angiogenesis models (outgrowths of aortic rings obtained from LHCGR-wild type and knockout mice or endothelial and mural cell proliferation and migration assays), Berndt et al. [21] demonstrated that hCG-H displayed a potent angiogenic effect by interacting with TGFβR, specifically by TGFβRII due to elimination of the hCG-H induced angiogenic effect by SB431542, the antibody against TGFβRII. Furthermore, this hCG-H-induced angiogenic stimulation was confirmed by an independent LHCGR-mediated mechanism, which demonstrated that it persisted in endothelial cells obtained from LHCGR-knockout mice [21]. This finding demonstrates a new paracrine interaction between trophoblast-secreted hCG-H and endothelial cell expressed TGFβRII, which contributes to angiogenesis crucial for placental development. This paracrine interaction has been shown to be mediated by activation of Smad 2 signaling in endothelial cells [21]. Worth considering is the observation by Koistinen et al. [52], claiming that the significant EGF contamination in hCG preparations used in studies by Berndt et al. [21], may contribute to TGFβR activation, partially disproving the role of hCG-H in activating TGFβR. Therefore, future studies are required to assess direct binding of hCG-H to TGFβR by confocal microscopy and/or electron microscopy using co-localization experiments following double immunostaining in the presence or absence of blocking antibody against EGF.

Similarly, co-immunoprecipitation performed in the same study [21] revealed binding of hCG-H to TGFβRII expressed by the Jeg-3 cells, a human choriocarcinoma (trophoblastic) cell line. Moreover, several groups implicated hCG-H in promoting growth and invasion of placental and germ cell malignancies through the TGF-β signaling pathway by utilizing potential autocrine interaction(s) [53,54]. This interaction between hCG-H and TGFβRII is further supported by structural similarity between hCG-H and TGFβ as they share a unique four-peptide cysteine knot structure identified in several cytokines that collectively form the cystine knot growth factor family [1,11,55]. Extra-sugar chains in hCG-H may prevent complete folding of the heterodimer, thereby exposing this cysteine knot structure and enabling binding of hCG-H to TGFβR. Thus, first trimester trophoblast apoptosis is reduced and EVT invasion is enhanced as a result of upregulation and/or activity of metalloproteinases [14,56]. Additionally, although hGC has been shown to block tissue inhibitor of metalloproteinases 1, 2, and 3 in decidualized endometrial stromal cell cultures [56], further studies are required to clarify whether this hCG effect on decidualized endometrial stromal cells involves LHCGR or TGFβR or both.

Moreover, hCG contributes to immunomodulation at the maternal-fetal interface by enhancing indoleamine 2,3-dioxygenase activity in dendritic cells, which reduces T-cell activation and cytokine production. hCG also induces recruitment Treg cells as well as their immune-suppressive capacity. Additional studies demonstrated that hCG stimulates proliferation of uterine NK cells and inhibits cytotoxic activity of circulating NK cells [6,7,57]. These hCG-mediated immune-modulatory actions are likely to facilitate implantation and trophoblast invasion by contributing to maternal immune tolerance against the semi-allogenic embryo.

## 4. Metabolism of hCG

Circulating hCG is metabolized primarily by the liver with approximately 20% of circulating hCG excreted by the kidneys [8]. During excretion, a major portion of hCG is degraded to subunits dominated by the β-core fragment (hCGβcf) [8,58]. In early pregnancy, levels of hCGβcf in urine are low [10], while in the second trimester, approximately 80% of immunoreactive urinary hCG levels consists of hCGβcf [59]. Wehmann et al. [60] and Korhonen et al. [61] studied clearance from the circulation of both endogenous hCG and injected purified hCG. The half-life of injected purified hCG conformed to a biphasic pattern (rapid phase: 5–6 h and slower phase: 24–33 h) [60,62], whereas that of endogenous hCG measured after a term pregnancy proved to be triphasic (3.6, 18, and 53 h) [61]. After term pregnancy or an abortion, hCGβ disappears more slowly than dimeric hCG (1, 23, and 194 h) [61]. Moreover, endogenous hCGα is metabolized faster than hCGβ, however, these half-lives are nevertheless longer than those observed after injection of purified hCGα (0.1–0.22 h and 1.2–1.3 h) [61,63]. The difference in half-lives between injected and naturally-occurring hCG may reflect partial denaturation to more rapidly metabolized forms during the purification process, whereas glycosylation may account for the slower metabolism of endogenous free subunits [10]. The extent of hCG glycosylation dictates molecular charge, such that more acidic isoforms exhibit a longer half-life in vivo, thereby governing clearance rate [64]. Metabolic clearance rates of deglycosylated, hCGβcf, and desialylated hCG occur faster relative to hCG, with the highest degree of acceleration observed for desialylated hCG [65,66].

## 5. Measurements of hCG Levels

In 1972, a rabbit antiserum specific for hCG was used to develop a radioimmunoassay to measure hCG in the presence of LH [67]. Currently, virtually all commercial assays for hCG are based on the “sandwich principle” that employs either monoclonal antibodies or monoclonal antibodies and polyclonal antiserum in combination. Basically, a monoclonal anti-hCG antibody is fixed to a solid phase to capture dimeric hCG, whereas a second monoclonal antibody/polyclonal antiserum is conjugated with a signaling agent (dye, radioactive material or enzyme for spectrometric/luminescence detection) and reacts with a distal site on the hormone to allow detection and measurement of captured hCG [68].

Antigenic regions on hCG have been extensively defined and monoclonal antibodies with known epitope specificity have proven instrumental in helping to design assays for each specific form of hCG [69,70,71,72,73]. These studies ascertained lack of cross-reactivity with LH [74]. In intact hCG, five epitopes can be discerned on hCGα (α_1_–α_5_) and seven on hCGβ (β_1_–β_5_, β_8_ and β_9_). Among these epitopes, β_2_ and β_4_ are both specific for hCG, hCGβ and hCGβcf, whereas antibodies to β_3_ and β_5_ also recognize LH. Two well-defined epitopes, β_8_ and β_9_, located on the CTP (absent on LH), are specific for hCG and hCGβ. Therefore, antibodies recognizing these β_8_ and β_9_ epitopes are used in many commercial assays [10,12]. Serum samples are preferred for quantitative hCG determinations, whereas urine samples are primarily used for pregnancy tests and to identify false-positive results in serum samples. Because both hCG and hCGβ may be present in serum, serum assays are usually designed to measure their combined levels. Assays detecting combinations of hCG, hCGβ, and hCGβcf are advantageous for the measurement of hCG immunoreactivity in urine [75,76].

False positive hCG results frequently arise from heterophilic antibodies that cross-react with immunoglobulins and may lead to potentially harmful interventions and other serious consequences. Mouse IgG, which blocks interference by heterophilic antibodies, is added to assays, but frequently at elevated/excess concentrations that result in falsely elevated results. In the absence of evidence of the presence of cancer, elevated serum hCG levels should be confirmed by repeat assay(s) in the presence of a blocking antibody, or by an alternative method, and/or hCG measurements in urine [12]. Familial hCG syndrome, a rare inheritable condition occurs in men and women [77] with a prevalence estimated to be 1:60,000. Affected family members produce a mutated hCG form with multiple alterations in the CTP region, which result in persistently elevated hCG levels (10–200 IU/L) that cause suspicion of pregnancy or cancer [77]. Therefore, confirmation of serum and urine hGC levels with different assays in combination with clinical observations is required to avoid unnecessary intervention.

## 6. Clinical Applications of hCG

### 6.1. hCG Measurements in Normal and Abnormal Pregnancies

Both diagnosing and monitoring pregnancy can be achieved using assays that recognize either hCG alone, or together with hCGβ. In men and non-pregnant women, hCG is produced by the pituitary and present at low serum levels [78]. In cycling women, the upper reference limit is 3 IU/L, which may increase to 6 IU/L in menopausal women [78]. However, values as high as 16 IU/L have been observed as a consequence of both differences in calibration and sensitivity among various assays [79]. In viable pregnancies, a median hCG concentration of 126 IU/L is observed 12 days after embryo transfer, whereas levels below 76 IU/L are associated with early pregnancy loss [80]. Approximately 20–30% of all pregnancies fail within days after implantation [18,81,82]. Compared with an ongoing pregnancy, a failing pregnancy is generally associated with lower serum HCG levels, which gradually turns into a decrease [83].

While hCG-H is well documented as a marker of early pregnancy, it is also proposed to be a better predictor of a viable pregnancy compared to hCG because failing pregnancies have been shown to produce minimal hCG-H [19,81]. A threatened abortion or a pregnancy of unknown location can be accurately monitored by serial measurements of serum hCG levels. Although an increase in serum hCG levels varies among pregnancies, its exponential increase predicts doubling of serum levels within ~1.5–2 day intervals to confirm a viable pregnancy [10]. A study by Barnhart et al. [84] evaluating hCG levels in symptomatic patients experiencing pain or bleeding after in vitro fertilization (IVF) determined that the slowest or minimal rise for a normal viable intrauterine pregnancy was 24% at one day and 53% at two days. More recently, a rise of 35% over 48 h was proposed as the minimal increase consistent with a viable intra-uterine pregnancy (IUP) [85].

No single reliable method exists to characterize the pattern of serum hCG change in women with a pregnancy of unknown location (PUL) who are subsequently diagnosed with an ectopic pregnancy [86]. A study by Korhonen et al. [87] reported a 1.5 days delay in the increase in serum hCG levels indicating that implantation is delayed. Noteworthy, is that a small percentage of ectopic pregnancies also demonstrate a clear and small rise in hCG levels. Clinically, the gold standard for diagnosis of a patient with PUL are measurements of hCGβ levels above the discriminatory zone (1500–2000 mIU/mL) without sonographic evidence of an intrauterine pregnancy. An intrauterine sac associated with hCG levels above the discriminatory zone reliably indicates an intrauterine pregnancy, but the absence of an intrauterine sac in conjunction with hCG values above this level suggests either a missed abortion or an ectopic pregnancy. Nevertheless, hCG values below the discriminatory zone combined with the absence of an intrauterine sac lacks diagnostic significance. A viable intrauterine pregnancy may be accompanied by an hCG rise of <50%. However, an increase in serum hCG levels of <35% is considered to be a safer definition of non-viability in a patient with a probable/likely ectopic pregnancy, especially when methotrexate administration is an option [85].

### 6.2. hCG, a Potential Biomarker for Preeclampsia

Preeclampsia is a syndrome affecting 5–10% of all pregnancies after 20 weeks gestation and is a major contributor to perinatal mortality and morbidity [88]. Currently, delivery of the placenta is the only definitive and effective treatment for preeclampsia. Abundant evidence indicates that the underlying pathology of preeclampsia occurs in the first trimester [89]. Therefore, early diagnosis during placentation may provide novel therapeutic options or may even prevent preeclampsia occurrence. Of note, a recent systematic review and meta-analysis suggests that in patients exhibiting risk factors for preeclampsia, initiating low-dose aspirin treatment in the first trimester until delivery can prevent preeclampsia [90,91]. Furthermore, measurements of early pregnancy serum concentrations of various maternal- and placenta-derived factors such as interferon-induced protein-10 (IP-10), soluble fms-like tyrosine kinase-1 (sFLT), matrix metalloproteinases (MMPs) [92,93,94,95,96] may prove valuable in identifying women at risk, as the development of the placental vasculature is frequently impaired in preeclampsia [97].

Pregnancies complicated by preeclampsia display an over-abundance of non-invasive syncytiotrophoblasts accompanied by inadequate cytotrophoblast invasion [98]. Preeclampsia is frequently accompanied by low hCG-H serum levels during the first trimester of pregnancy (8–13 weeks) [99]. A recent study by Kalkunte et al. [100] found higher hCG levels in serum from preeclamptic pregnancies at term compared with serum derived from normal pregnancies. The pathogenesis of preeclampsia is associated with altered glycosylation patterns and/or presence of sialyl Lewis antigens on hCG, which impairs the recruitment and/or expansion of tolerance-inducing immune cell types [88,101]. The combination of hCG-H together with pregnancy-associated plasma protein A (PAPP-A) levels, maternal mean arterial pressure and parity were shown to predict early onset preeclampsia with an area under the curve (AUC) value exceeding 0.85 [99]. A meta-analysis of 4 studies [99,102,103,104] by Zhong et al. [105] evaluated first-trimester serum screening for 112,400 women in order to predict preeclampsia. Although the results showed no improvement in prediction, the detection rate of first trimester serum markers for early preeclampsia was observed to be better than that for late preeclampsia.

### 6.3. hCG, a Serum Marker for Down’s Syndrome Screening

Measurements of hCGβ levels in first trimester maternal serum proved to be extremely useful in screening for Down’s syndrome. Pregnancies complicated by Down’s syndrome are associated with elevated serum hCG and hCGβ concentrations. Recommended screening for trisomy 21 (Down’s syndrome) includes a combination of maternal age, fetal nuchal translucency (NT) thickness, maternal serum hCGβ and PAPP-A at around 11–13 weeks gestation [106]. However, for second trimester (15–22 weeks gestation) screening, hCG is combined with inhibin A, α-fetoprotein (AFP) and unconjugated estriol. To assess patient-specific risks for trisomy 21, a prior maternal age-related risk is multiplied by likelihood ratios determined from deviation of the measured markers from the respective expected levels [107]. Furthermore, several markers of Down’s syndrome (hCGβcf, hCGβ, and hCG-H) can be detected in maternal urine [108]. Among these, hCGβcf is the major metabolic product of hCG in maternal urine with second trimester levels increasing in pregnancies complicated by Down’s syndrome [108]. Urinary hCG-H levels are also elevated in affected pregnancies although fewer cases have been tested for hCG-H compared to hCGβcf levels. Maternal serum hCG assays remain the preferred test for Down’s syndrome screening versus urine hCGβcf or hCG-H due to the wide standard deviation of the urine markers and the significant heterogeneity that exists among studies [108].

### 6.4. hCG Is Crucial in the Diagnosis of Gestational Trophoblastic Disease

Gestational trophoblastic disease (GTD) is comprised of a spectrum of interrelated conditions originating from the placenta. Additional terms frequently used to refer to these conditions include gestational trophoblastic neoplasia and gestational trophoblastic tumor. GTD includes a variety of tumors ranging from complete and partial hydatidiform moles, invasive moles, gestational choriocarcinomas, and placental site trophoblastic tumors. Estimates of the incidence of various forms of gestational trophoblastic disease vary. In the United States, hydatidiform moles are observed in approximately 1 in 600 therapeutic abortions and 1 in 1500 pregnancies [109]. Approximately 20% of patients eventually develop malignant sequelae requiring administration of chemotherapy after evacuation of hydatidiform moles [110,111]. Trophoblastic tumors produce elevated serum concentrations of hCG compared to normal pregnancy, representing the most sensitive tumor marker available to diagnose these conditions [10]. Trophoblast tumors also produce high hCGβ levels, but usually at lower concentrations than hCG. Compared to other types of GTDs, placental site trophoblastic tumors frequently produce small amounts of hCG with 25% reported to be hCG negative [112].

The percentage of identifiable hCGβ positive trophoblastic tumors depends on the assays used for detection. Specifically, molar hCGβ concentrations >5% are strongly associated with aggressive GTD [113,114], with a rising proportion found to predict the development of chemotherapy resistance [115,116]. Assays that measure hCG and hCGβ together are primarily used to monitor patients with GTD. However, use of separate assays for hCG and hCGβ facilitate differentiation between benign and malignant trophoblastic diseases. Patients whose hCG levels increase after becoming undetectable are considered to have relapsed. In contrast, patients whose hCG levels remain elevated despite treatment are considered to have resistant disease. Relapse is usually detected on the basis of increasing serum hCG levels before the tumor is large enough to be detected by an alternative method. Before the advent of sensitive assays for hCG, cases of GTD associated morbidity and mortality were substantial. However, development of more sensitive assays and targeted therapies offer a promising future. Currently, the majority of women with malignant GTD can be cured and their reproductive function preserved.

### 6.5. hCG Use in Assisted Reproductive Technology

The mid-cycle LH surge is essential to achieve normal oocyte maturation and ovulation [10]. Partially- purified urinary hCG (surrogate for LH) preparations are administered to achieve final oocyte maturation and ovulation during controlled ovarian hyper-stimulation, and to facilitate correct timing of oocyte retrieval [117]. As a cautionary note, recent studies revealed that, upon binding to LHCGR, LH, and hCG, each triggers different intracellular signaling cascades (AKT, ERK1/2 MAPK, and β-arrestin 2) and steroidogenesis [39,42]. Similarly, hCG use in assisted reproduction may result in different responses than LH with respect to mature oocyte collection, embryo quality, implantation and pregnancy rate [118,119]. Moreover, Tesarik et al. [120] showed that hCG administration to recipients increased endometrial thickness on the day of embryo transfer and improved endometrial receptivity. Recently, recombinant-hCG (r-hCG) became commercially available and subcutaneous administration of 250 µg of r-hCG was found to be equivalent to, or at least as effective as, intramuscular 10,000 IU of urinary hCG in initiating final oocyte maturation [121]. In addition, administration of r-hCG is associated with significantly improved patient tolerance compared with urinary hCG administration [122].

## 7. Conclusions

**Figure 1:**
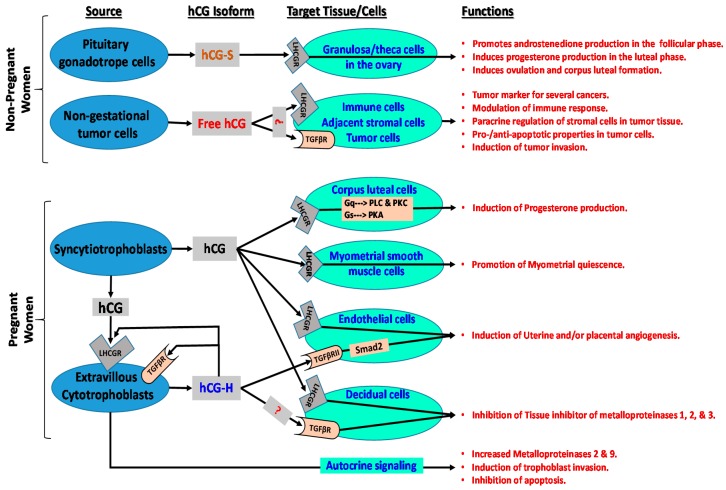
Cellular sources, targets, associated signaling cascades, and functions of various hCG isoforms in non-pregnant and pregnant women. LHCGR: luteinizing hormone/choriogonadotropin receptor; TGFβR: transforming growth factor beta receptor; ?: hCG may bind to relevant receptor in target cells; Smad2: similar to drosophila gene ‘mothers against decapentaplegic’ 2; Gq: heterotrimeric G protein subunit that activates phospholipase C (PLC)-associated protein kinase C (PKC); Gs: heterotrimeric G protein subunit that activates cAMP-dependent protein kinase A (PKA) signaling by activating adenylyl cyclase; hCH-S: sulfated hCG; hCG-H: hyperglycosylated hCG.

The hCG molecule is an extremely important multifaceted hormone involved in hormonal interactions of the fetal-placental-maternal unit, as well as neuroendocrine and metabolic changes that occur in the mother and in the fetus during pregnancy and at parturition, as well as pathophysiologic functions in non-pregnant women as summarized in Figure 1. Produced by a variety of organs, existing in various forms, and displaying vital biological activity, hCG is also involved in important clinical functions ranging from diagnosis and monitoring of pregnancy, early detection of pregnancy-related disorders, prenatal aneuploidy screening, detection of gynecological cancers, and treatment of infertility. A summary of the current literature is presented in Figure 1. However new cellular sources, molecular interactions as well as functions for this unique molecule are discovered continuously. Further research is needed to identify hCG receptor(s) and associated intracellular signaling cascades and to increase understanding of its role in achieving conception and in pregnancy-related disorders.
